# Future teachers' perception of the usefulness of SketchUp for understanding the space and geometry domain

**DOI:** 10.1016/j.heliyon.2021.e08206

**Published:** 2021-10-19

**Authors:** Enrique Carmona-Medeiro, Juan Antonio Antequera-Barroso, José María Cardeñoso Domingo

**Affiliations:** a Faculty of Education, Department of Didactics, Area of Didactic of Mathematic, University of Cádiz, Puerto Real, 11519 Cádiz, Spain; b Faculty of Teacher Training, Department of Didactics of Experimental Sciences and Mathematics, Area of Didactic of Mathematic, University of Extremadura, 10071 Cáceres, Spain

**Keywords:** Mathematics education, Educational informatics, Educational innovation, Teacher training, Geometry

## Abstract

This article reflects the opinion of future Early Childhood Education teachers at the *Universidad de Cádiz* on the usefulness and degree of satisfaction of SketchUp, a 3D modelling software programme, after they participated in a workshop for didactic-mathematical training. They had to use the software to design and model their ideal nursery school in 3D, supported by clearly stated and well-defined educational pillars. This study aims to ascertain the students' perceptions of the use of this resource with the intention of assessing its suitability to offer more appropriate initial training regarding mathematics education. It seeks to make the most of using the software programme and minimise the obstacles encountered. Opinions were collected from a sample of 203 students who responded to two questionnaires designed ad hoc. The results are organised around a SWOT analysis and show a satisfactory global evaluation.

## 1 Introduction

The circumstances of the COVID-19 pandemic, which we are currently experiencing worldwide (Hodges et al., 2020; Porlán, 2020; UNESCO, 2020), affect all areas of society, and generate important social, personal and economic difficulties. Higher Education has not been spared either, and has had to adapt fast to blended and on-line training contexts where the use of new technologies has been imposed as a non-negotiable need. However, this supervening situation offers us the opportunity to enquire about which ICT resources are potentially interesting for our training purposes. More importantly, it allows us to explore how these resources can be integrated to create an educational proposal that has a significant impact on the professional development of our students. Incorporating new technologies is also an opportunity for training in digital competences that future mathematics teachers should possess (Stein et al., 2020). Those competences can help them when incorporating ICTs in their future teaching and professional practice (Tokmak et al., 2013). The use of ICTs in themselves does not add value to the training process, it is the use the teacher makes of them that provides them with value. Training teachers in the use of ICTs is therefore key in the development of digital competences.

The ways of communication with and between students have changed completely and the classroom context makes the use of manipulative materials impossible. All this has entailed a deep reflection in search of new procedures to address, amongst many others, the following questions (Esteve Ruescas and Alsina, 2020): how to plan active learning activities that encourage enquiry and promote the development of the didactic-mathematics competence of students in on-line learning environments? How to replace the use of manipulative materials? How to ensure student motivation and performance? During those activities, how to manage and mediate, encouraging communication, discussion and negotiation between students and with the teacher?

Although we have tried to keep the meaning and educational value of the subjects intact, the process of reflection has led to a significant reconstruction of the activities, strategies and methodological actions we had been using in previous courses. To illustrate this reflective process, one of the activities implemented in the subject called *development of mathematical knowledge in Early Childhood Education*, taught on-line in the third year of the Early Childhood Education (ECE) degree, will be presented.

Said activity is organised in a workshop called *We build our ideal school,* in which we replaced the traditional use of manipulative material (Polydron, Geomag, Multicubes, etc.) by the use of the SketchUp design software. Our intention was to generate the need to coordinate logical-mathematical knowledge, as well as its learning and teaching, in a realistic context linked to the students' future professional activity.

In this article, we focus on the evaluation the students carry out of the usefulness and degree of satisfaction of the use of the SketchUp software for their training in the space and geometry domain (SGD) through implementing the workshop.

## 2 Literature review

The widespread use of new technologies and the search for innovative learning environments that connect with the current interests of students has led to the use of dynamic geometry programmes such as SketchUp. They are aimed at encouraging the development of spatial-geometric skills in the different educational stages (Nurwijayanti et al., 2019; Lee and Kim, 2013; Turgut and Uygan, 2015; Ibili, 2019; Poonpaiboonpipat, 2021).

The results obtained in research studies on the educational potential of SketchUp show that, in most cases, the software helps improve the visual and spatial skills of those students who have used it as opposed to students who have not used it (Kurtulus and Uygan, 2010; Toptaş et al., 2012; Dolenc and Aberšek, 2012; Turgut and Uygan, 2015; Chou et al., 2017; Wahab et al., 2018; Williams and Capraro, 2020; Šafhalter et al., 2020; Jaelani, 2021). However, not all the authors find significant differences between the groups (Erkoç et al., 2013), and some consider that success lies in teacher preparedness when applying the software in the classroom (Benzer and Yildiz, 2019; Poonpaiboonpipat, 2021).

Regarding the understanding of the SGD, the research findings indicate that the software facilitates the understanding of the position and dimension of objects and their relationship with the space surrounding them (Dolenc and Aberšek, 2012; Panorkou and Pratt, 2016; Uygan and Kurtuluş, 2016; Nurwijayanti et al., 2019), as well as the relationship of space and geometry with nature and/or with geography (Galani, 2015).

Other advantages identified in the research literature on the educational use of software are the work environment (Dolenc and Aberšek, 2012; Lee and Kim, 2013), dialogue, debate and negotiation among peers (Panorkou and Pratt, 2016; Capraro et al., 2018), and the ease of obtaining realistic results through using colours and textures for the design of the different elements (Ramadhanty and Handayani, 2020).

Regarding the software manageability, the results show, in most cases, that its use can be learnt quickly and easily (Erkoç et al., 2013; Chou et al., 2017), although some research studies highlight its use takes time, and can be difficult to understand (Uygan and Kurtuluş, 2016).

## 3 Implementation of the workshop

The workshop *We build our ideal school* is proposed in terms of a contest, stating the different parts to be completed in a constructive and argumentative manner in small working groups. The starting point of the workshop is the proposal to design a school that, justified from its own perspectives, can host the educational experiences the students consider to be the most powerful for child development. They are generated to analyse and reflect on the idea that space and its organisation also educate (Arnaiz et al., 2011).

Theoretical references imply considering a space that configures an open, communicative, welcoming, stimulating nursery school (Sánchez and González, 2016). It should be built from well-defined and well-structured educational pillars (Cardeñoso and Azcárate, 2002; Brenneman et al., 2009; García-González and Schenetti, 2019) that are anchored in a genuine trust in children, their values, abilities, and possibilities to grow and become happy and productive human beings for society (Carmona and Huitrado, 2013). The design should reflect the professional identity of the authors (Porlán, 2020).

**Figure 1:**
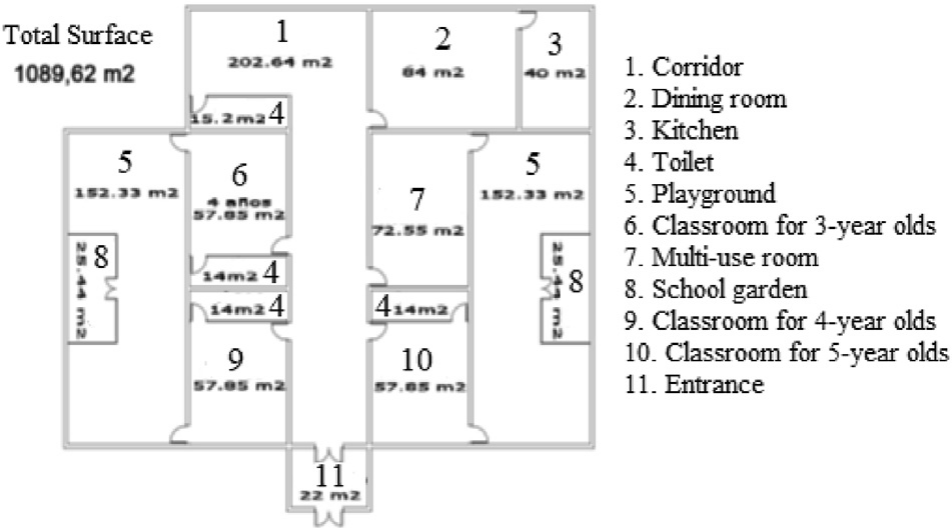
Drawing of the design of the school by one of the groups of students (Gr10).

**Figure 2:**
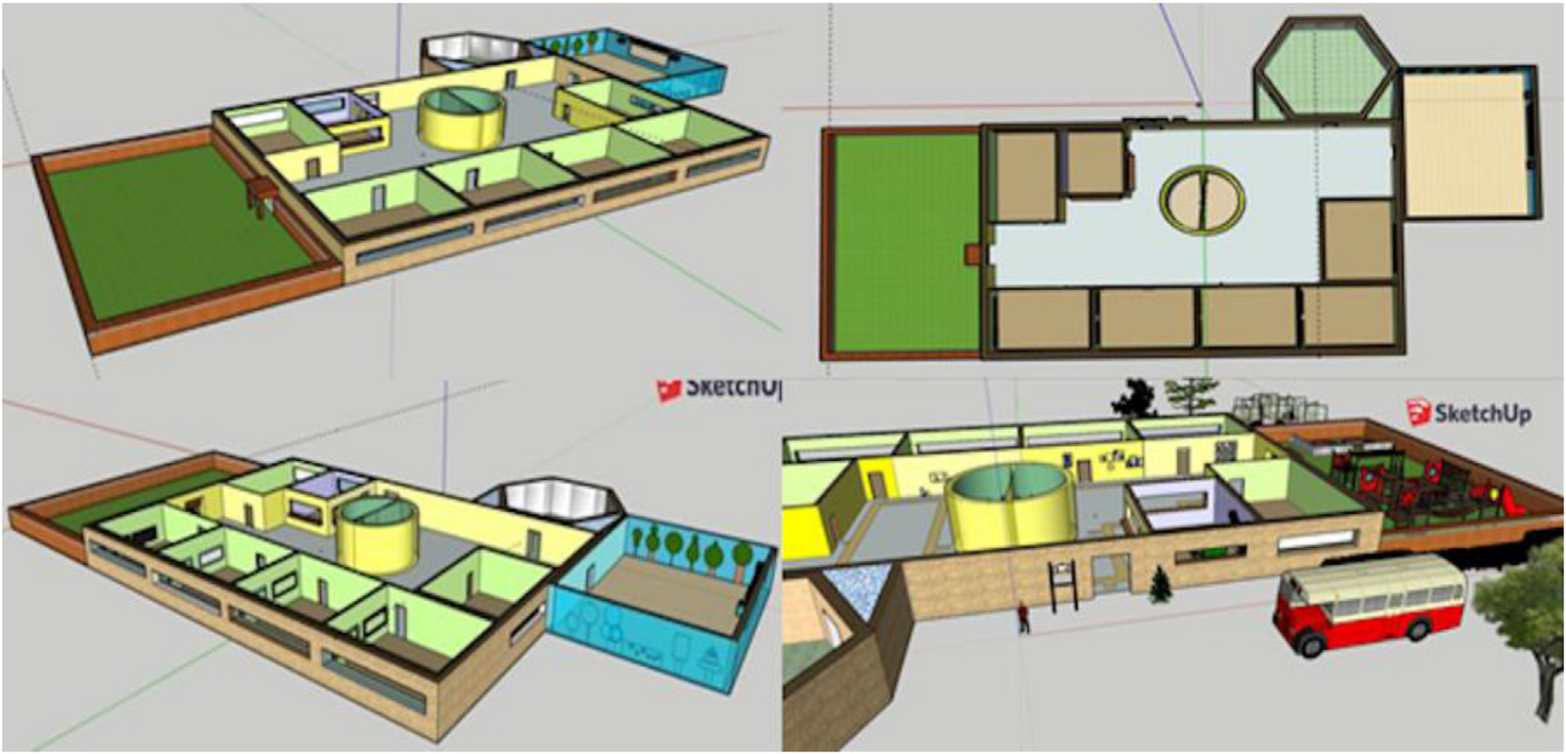
Example of the design of a school using SketchUp (Gr18).
• Next, the students have to defend the strengths of their design and justify the decisions made by the group in their design.
• As a third step, a meta-reflection exercise on the mobilised mathematical knowledge is required, emphasising the mathematical knowledge characteristic of the SGD, from reflecting on the process performed.

Implementing the workshop is not only an opportunity to develop digital competence. The workshop must also provide the opportunity to build up the mathematical knowledge of reference, the space and geometry domain. For this purpose, a sequence of tasks is designed for the different groups to work on throughout the workshop:

• The first task is designing the school, which involves both sketching out a plan (Figure 1), and turning it into a 3D model, using the SketchUp software (Figure 2).

At the end of the workshop, the students will be asked to evaluate the use of the software and the knowledge mobilised.

### 3.1 Description of the SketchUp software

The impossibility of using manipulative materials for the development of the training workshop on the SGD opened the door to explore different design and 3D modelling software programmes. Opting for SketchUp (managed by Trimble) is justified by the availability of a basic 3D modelling course with SketchUp Make hosted by and available in Spanish at *Universidad de Cádiz* (UCA) (http://cursosenabierto.uca.es/curso-basico-de-modelado-3d-con-sketchup-make/).

A free version can be obtained from the official software page (https://www.sketchup.com/es) to be able to start working from the website itself. This version offers 3D modelling on the web, the possibility of viewing the creations on the mobile and cloud storage of up to 10Gb to be able to share the work done. Their definition of the programme is that it is a software based on 3D modelling in which edges and sides can be modified to obtain the desired result from a 2D image.

**Figure 3:**
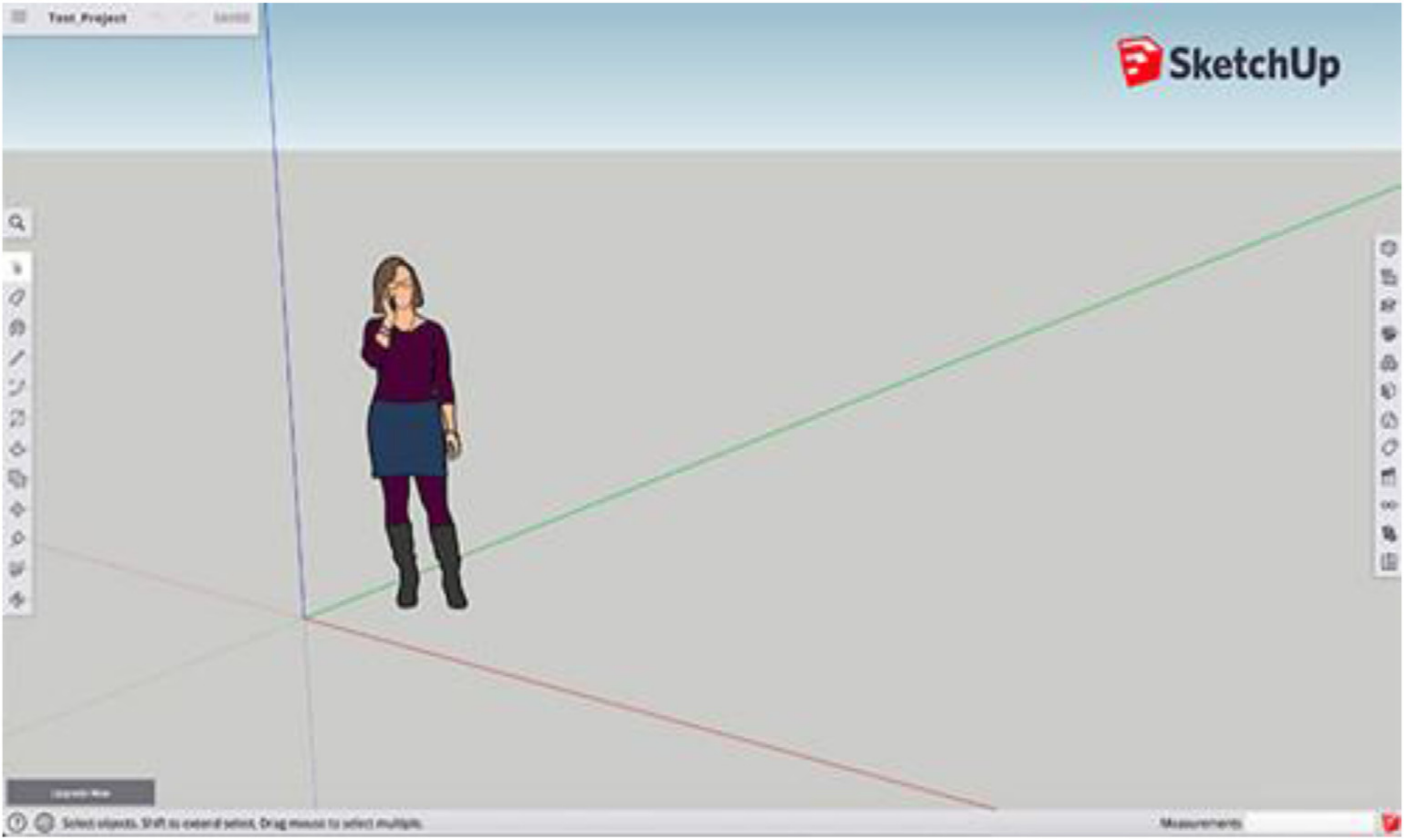
Image of SketchUp's initial interface (obtained from the SketchUp web page).

Figure 3 shows the interface in which to start designing, once registration and configuration have been completed. On both sides of the image, there are toolbars that allow building and designing the structure(s) proposed in the sketch, as well as all the architectural details considered appropriate for its their development.

The toolbar on the left serves to initiate the building process, starting from a pre-established geometric figure, or lines joined together, which represent the germ for the construction of the final structure. In this same bar, there is another tool that allows transforming a 2D structure into a 3D one and, from here, it enables working on the different details, such as creating the openings in the different rooms the structure includes. The tool also enables monitoring the different perspectives from which to check the progress of the structure(s) created. The bar on the right of the image is used for the design of the structure built with the bar on the left. It is possible to use the designs provided by the software itself. They can be activated in this toolbar or can be adapted to the needs of what is imagined in the design proposal of the different groups' ideal school, as a large number of materials and textures are available.

It is also possible to analyse the dimensions of the different structures or objects created thanks to the tool that appears in the lower right part of the image. It allows checking if the chosen dimensions are appropriate for both the objects and the different rooms and the relationship between them. Finally, it should be noted that each button on the toolbar has a help option associated to it that shows its function.

Our working hypothesis is that SketchUp enables analysing the relationships that can be established between the interior and exterior elements of the school as well as with its location in a town, a forest or near the beach, in order to take advantage of all the resources present in the area where the ideal school has been built (Schenetti et al., 2015). Its use therefore encourages a study process on the elements and relationships that take part in the design proposed in the workshop and promotes processes of reflection in the students on the spatial and geometric relationships that affect their understanding.

## 4 Methodology

Taking into account the criteria established by Latorre et al. (1997), we understand that we are facing a descriptive-interpretive study (Aguirre and Jaramillo, 2015) aimed at knowing the perceptions of a specific group of future ECE teachers. We decided to use mixed methods and techniques. Mixed methods combine the quantitative and qualitative perspective in the same study. This allows analysing the characterisation and interpretation of the data in-depth when research questions are complex Barrantes (2014); Flick (2012); Creswell and Plano (2011). It thus enables us to, on the one hand, characterise a singular reality by analysing the responses and their implications and, on the other hand, to obtain a more general image of the group of students because of the large number of participants. We have therefore approached the students' perceptions by using a questionnaire complemented with a Likert-type scale questionnaire (Sullivan and Artino, 2013).

### 4.1 Research question and objectives

As mentioned earlier, the COVID-19 situation has given us the opportunity to explore the use of the SketchUp software for the first time in a non-face-to-face educational context of didactic-mathematical training.

Once the workshop was implemented, it was necessary to evaluate the proposal from different angles. In this paper, we focus on understanding how the students perceived the use of the software. By doing so, we try to elucidate the added value that its use has given to the workshop, as well as the obstacles and difficulties it has generated.

The purpose of this study is to discern, in a logical and reasoned manner, the possibility to continue using it in future courses, and making possible improvements. The research question asked is the following: What usefulness have the students observed in the use of the SketchUp software for the understanding of the SGD?

To answer this question, the following objective was formulated: Identify, describe and understand the usefulness students perceive in the use of SketchUp software for understanding the SGD in the development of a training workshop.

This general objective is specified in three sub-objectives:

• Identify, describe and understand the strengths and weaknesses students perceive in the use of the SketchUp software for the understanding of the SGD
• Identify, describe and understand the opportunities and threats students perceive in the use of the SketchUp software for understanding the SGD
• Assess the degree of satisfaction the use of the software has provided the students with

### 4.2 Participants

The sample consisted of a total of 206 students in the third year of the ECE degree at UCA, organised into 37 groups (Gr1-37), and obtained the participation of 203 of them in a process that has at all times ensured both the anonymity and confidentiality of the information.

This study ruled out the possibility of taking gender variables into account because of the particular homogeneous distribution of the ECE degree, where over 90% of the total number of students are female. Likewise, the option of considering age was rejected, since all the students are approximately the same age.

### 4.3 Data collection procedure and instruments

The information related to the perception of the students (Cabero-Almenara et al., 2018) about the usefulness of the SketchUp software for learning the SGD was collected through an ad hoc questionnaire including open questions, structured around the SWOT analysis technique. Applying the SWOT technique to virtual teacher training allows students to reflect on and assess the specific problems of integrating ICTs in classroom activities (Colás and Pons, 2004).

The following questions were asked:

1. In your opinion, what advantages (strengths) has the use of the SketchUp software offered for the design of your ideal school? Has it favoured the understanding of any aspect related to the SGD?
2. In your opinion, what disadvantages (weaknesses) has the use of the SketchUp software presented for the design of your ideal school? Has it made it difficult to understand any aspect related to the SGD?
3. For which other activities could the SketchUp software be used (opportunities) in order to contribute to a better understanding of some aspect of the SGD?
4. For which other activities or situations could the use of the SketchUp software be a problem (threats)?

In line with the work of Azcárate and Cardeñoso (2011), we believe this technique is appropriate for the purpose proposed, since it will allow us to specify and cross-check the evaluation of the strengths and weaknesses (internal analysis) of the proposal with the threats and opportunities (external analysis) that involve the use of the software for learning the concepts of the SGD.

To assess the degree of satisfaction of the students with respect to the use of the SketchUp software, an online questionnaire developed and validated by Coll et al. (2008) was used. The satisfaction variable is measured through four items, three of them with a scale of five responses where 1 = Not at all satisfactory and 5 = Very satisfactory, and one is dichotomous.

The following questions were asked:

I. Taking into account the purpose of the workshop held, your overall evaluation of the approach and development of the experience is:
II. Taking into account the purpose of the workshop held, your overall evaluation of the use of the SketchUp software for the development of the experiment is:
III. How would you evaluate the way the teacher has led the workshop?
IV. If next term/year you were to take part an activity that required designing objects or spaces in 2D or 3D and you were free to choose, would you opt for the SketchUp software?
V. What other feedback would you like to provide?

In the first and third questions, the students are asked to express their degree of satisfaction with respect to the experience of the workshop and the work of the teaching staff in global terms. In the second, they are asked about the degree of satisfaction with the use of the software in the workshop. The fourth one asks about their position on whether or not they would choose to use the software again. The questionnaire ends with an open question for the students to express other opinions of interest.

The questionnaires to collect the information related to the perception of the students about the usefulness of SketchUp and the information related to the degree of satisfaction with respect to its use were applied in December 2020 by means of online questionnaires included in the Moodle platform where the subject was taught.

### 4.4 Data processing and analysis

To process the data obtained from the questionnaire, a procedure of direct interaction with the data was used to properly select the meaningful information units.

The different answers provided by the students were read and analysed as a whole. The relevant data for the purpose of our study were selected from the students' reports, and are described below. The unit of information is understood as the unit of significance to be coded, which is highly variable in nature and size (Bardin, 1986, p. 79).

To perform the content analysis of the responses offered by the participants in the questionnaire, we freely coded the comments collected in each of the SWOT elements. This allowed us to build emerging categories and group the students' responses into those categories following an iterative coding process. During the coding process, through grouping by units of meaning, different categories and indicators emerged that were refined during and after the subsequent analysis (Strauss and Corbin, 1990), following the meaning condensation method proposed by Kvale (1996). A total of 9 categories (C1–C9) (Table 1) and 34 indicators were constituted among the four elements of the SWOT analysis (S_i_, W_i_, O_i_ and T_i_), where S_i_, W_i_, O_i_ y T_i_ represent the i-th indicators related to strengths, weaknesses, opportunities and threats. They belong to both internal and external factors, as will be shown in the results section.

**Table 1:** Emerging categories related to strengths, weaknesses, opportunities and threats.

Categories related to strengths	Categories related to weaknesses
C1. Software management	C1. Software management
C2. Student motivation and involvement	C2. Student motivation and involvement
C3. Spatial understanding	C6. Limitations of the software as an educational resource
C4. Geometric understanding	
C5. Mathematical communication	
Categories related to opportunities	Categories related to threats
C7. Innovations in the area of mathematics	C8- Professional development
C8. Professional development	C9. External risks

Within each category, several kinds of information units were distinguished. This enabled formulating different indicators in which to group those units. The indicators reflect the different perceptions formulated by the students in their reports. They allowed us to classify the expressions the students used to identify, and describe their perception of the usefulness of the SketchUp software to facilitate the understanding of the SDG.

Regarding the Likert scale questionnaire, the responses were processed following the algorithm proposed by Kvon et al. (2018) for a validated instrument.

## 5 Results

We here present the results organised for their subsequent discussion.

### 5.1 Perception of the degree of satisfaction of the students about the use of the SketchUp software (Likert scale questionnaire)

First, the general results obtained for each of the questions are shown, taking into account the students' responses to questions 1, 2 and 3 (Table 2), and the percentage distribution of the responses.

**Table 2:** Results of the students' responses to the questions posed.

Responses	Points per response	Questions		
		I	II	III
Very satisfactory	5	23 (11.3%)	10 (4.9%)	14 (6.9%)
Quite satisfactory	4	127 (62.6%)	99 (48.8%)	114 (56.2%)
Neutral	3	37 (18.2%)	50 (24.6%)	45 (22.2%)
Slightly satisfactory	2	12 (5.9%)	27 (13.3%)	23 (11.3%)
Not at all satisfactory	1	4 (2%)	17 (8.4%)	7 (3.4%)
**Number of responses**		203	203	203
**Total**		762	667	714

The results in Table 2 show high student satisfaction with respect to the overall experiment (question I) of their experience in the workshop, combining the positive responses (“very satisfactory” and “quite satisfactory”). The global score of the experiment was high for 73.9% of the students, and low for a significantly small percentage of the students (7.9%).

Regarding the global evaluation of the students about the use of the SketchUp software (question II) in the development of the workshop, the results of Table 2 show moderate student satisfaction, combining the positive responses (“very satisfactory'” and “quite satisfactory”). The use of the software obtained a high score for 53.7% of the students, while, for a relatively significant percentage of the students, combining the negative responses (“slightly satisfactory” and “not at all satisfactory”), the score was low (21.7%).

As far as the students' evaluation of how the teacher has led the workshop is concerned, the results in Table 2 show considerable student satisfaction, combining the positive responses (“very satisfactory” and “quite satisfactory”). The teacher's guide obtained a high global score for 63.1% of the students. In contrast, a modest percentage of students, combining negative responses (“slightly satisfactory” and “not at all satisfactory”) obtained a low score (14.7%).

Finally, the results obtained in the dichotomous question: If next term/year you were to participate in an activity that required designing objects or spaces in 2D or 3D and you were free to choose, would you opt for the SketchUp software? 54.6% said they would, and 45.4% said they would not.

### 5.2 Perception of the usefulness of the SketchUp software for learning the SGD (SWOT analysis)

After completing the questionnaire and collecting data on the students' perception of the usefulness of the software for learning the SGD in the workshop, we proceeded to analyse and reflect on the results obtained, identifying both the internal factors (strengths and weaknesses) and external factors (threats and opportunities) the use of the software in the workshop meant to the students.

An analysis of the results obtained interpreting how the students perceived the use of the SketchUp software is provided below. It aims to identify the obstacles and difficulties that derived from its use.

Although the results show high student satisfaction (73.9%) with respect to the approach and development of the workshop, the results related to their evaluation regarding the use of SketchUp reveal a significant drop in student satisfaction (53.7%) as far as the overall evaluation of the workshop is concerned. This substantial difference is partly related to the use of the software, since 21.7% of the students is not satisfied with its use.

This decrease regarding the use of the software may be related to the weaknesses and threats the students mentioned in their responses regarding the use of the software. This information will help us interpret the significant change observed in the global evaluation of the use of the software and identify the obstacles and difficulties that arose from its use.

The weaknesses and threats identified in the responses of the students are grouped together in the table below. They include the harmful elements of the software, such as those that conditioned the learning of the SGD throughout the workshop (Table 3), as well as the frequency of mention (n_i_), and the percentage distribution.

**Table 3:** Categories and indicators associated with the low satisfaction of the use of the software.

Weaknesses, associated with:	n_i_	%
**C1**. S**oftware management**	84	-
**D**_**1**_. Complicated, difficult	84	41.4
**C2. Student motivation and involvement**	78	-
**D**_**2**_. The software is boring, tedious and lacks usefulness	17	8.4
**D**_**3**_. No face-to-face classes: lack of peer interaction	24	11.8
**D**_**4**_. Lack of immediate feedback from the teacher	37	18.2
**C6**. **Limitations of the software as an educational resource**	56	-
**D**_**5**_. Free version of the software is limited	26	12.8
**D**_**6**_. Aimed at other professionals	21	10.3
**D**_**7**_. Requires installation	9	4.4
Threats, associated with:	n_i_	%
**C9. External risks**	169	-
**A**_**1**_. Digital gap	26	12.8
**A**_**2**_. Impossibility of using the software including all its features without purchasing its license	57	28.1
**A**_**3**._ The online version of the software generates distrust.	31	15.6
**A**_**4**_. Existence of other more attractive programmes	55	27.1
**C8- Professional development**	33	-
**A**_**5**_. This software is not appropriate for the teaching profession	33	16.3

It is observed that the most mentioned weaknesses correspond to the categories of *software management* (**C1**) and to *student motivation and involvement* (**C2**), while the most mentioned threat corresponds to the *external risks* (**C9**) category.

Likewise, the most mentioned weaknesses correspond to the *complicated, expensive* **(D**_**1**_**)** indicator, which refers to the category of *software management* **(C1)**, and to the *lack of immediate feedback from the teacher* indicator **(D**_**4**_**)**, which refers to the *motivation and involvement of students* **(C2)** category. This weakness may be related to the difficulty of interaction between students and teachers generated by the current situation. It is an aspect to take into account in future designs. The most mentioned threats correspond to the *impossibility of using the software including all its features without purchasing its license* **(A**_**2**_**)** and the *existence of other more attractive programmes* (**A**_**4**_) categories, which belong to the *external risks* category **(C9)**, aspects that are external to the design of the workshop and can hinder its development.

Regarding the *software management* (**C1**), 41.4% of the students states that its use is *complicated, difficult* (**D**_**1**_) and that mastering the different tools requires considerable time for reflection and practice. One of the groups mention this: “*It has taken us a lot of time and effort to learn to use the programme, and we have had to consult several tutorials*” (Gr36).

With respect to *student motivation and involvement* (**C2**), 8.4% of the students considers that the software is *boring, tedious and lacks usefulness* (**D**_**2**_), as shown in the following statement: “*Using the programme has been a waste of time… we haven't learned anything important and I don't think we'll ever use it again*” (Gr22).

12.8% points out that the impossibility of using all the software features in the free version (**D**_**5**_) lowers the quality and makes the 3D modelling of the school lose realism and attractiveness, diminishing the students' initial expectations and altering their assessment of the software as an educational resource (**C6**). In this sense, 28.1% of the students considers that not being able to enjoy all the benefits of the software without purchasing the license (**A**_**2**_) is a threat (**C9**) and an important reason not to use it in the future, as shown in the statement of one of the groups: “*It is a pity not to be able to use many of the tools, after all the effort it is a nuisance not to be able to finish all the details to our liking… If we had to use another programme in the future, we would look for one without limitations of use*” (Gr13).

**Figure 4:**
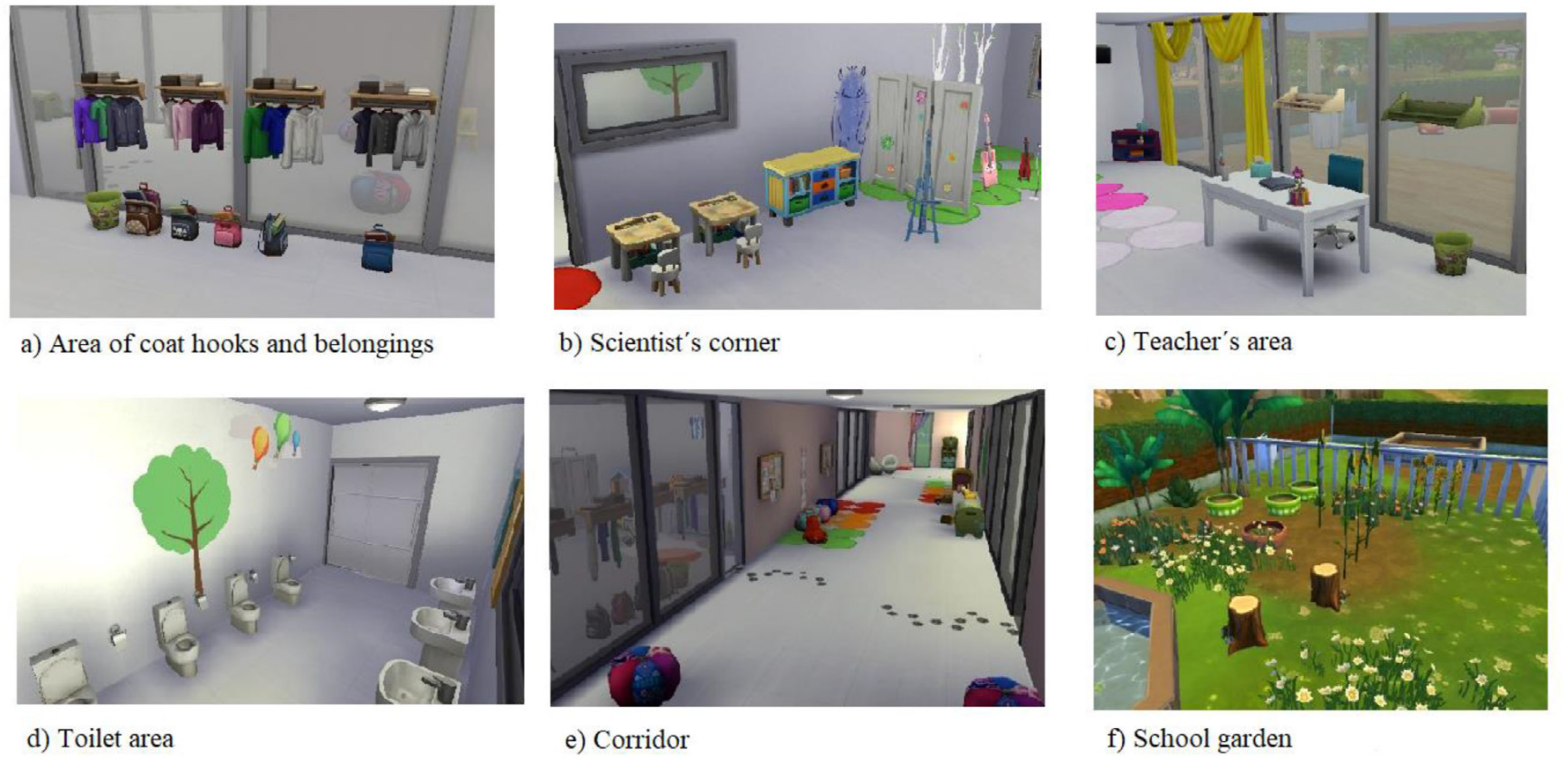
Images of the spaces of a school built with The Sims (Gr2).

Regarding the results offered by the software, the students consider as a threat the fact that the result does not conform to what they expect (**A**_**2**_), and therefore causes disappointment with their work or the result prevails over the knowledge they want to work on, in this case the space and geometry domain. Likewise, 27.1% does not consider the future possibility of using SketchUp again, and points out the existence of other more attractive and well-known programmes (**A**_**4**_) such as Second life, The Sims, SimCity and others, with which they think they will be able to construct a more attractive and detailed result more easily that would provide added value to the school modelled in 3D. One of the groups that built the school in parallel with The Sims (Figure 4) expressed the following: “I can do many more things with The Sims, there is an immense repertoire to build whatever I want, I do it faster and the finish is spectacular, much more realistic than when using SketchUp” (Gr2).

With reference to the didactic use of the software (**C6**), 10.3% claims that SketchUp would be interesting for professionals or students of architecture or engineering degrees, but that it is by no means appropriate for teacher training students (**D**_**6**_). Therefore, 16.3% of the students points out that the software is not suitable for the teaching profession (**A**_**5**_) and they consider it a threat, as observed in the following statement by one of the groups: “*The software is aimed at engineers and architects ... We do not think it is appropriate for teacher training students*” (Gr17).

As for the teaching management of the workshop, 18.2% of the students highlights that the lack of immediate feedback from the teacher (**D**_**4**_) to meet the needs related to software management and the demands of the workshop, together with the difficulty of working from home without direct contact with classmates (**D**_**3**_), pointed out by 11.8%, made the experience more difficult and affected their motivation and involvement (**C2**). It is reflected in the following statements: “*In the face-to-face format, everything would have been easier… We wasted a lot of time waiting for the teacher to help us solve difficulties regarding the management of the software*” (Gr21) or “*Doing group work without being together has not been easy, we would have finished long before if we had been working in the classroom with the teacher present*” (Gr33).

The free online version of the software does not generate trust (**A**_**3**_) according to 15.6% of the students. They also point out that several plug-ins need to be installed and that the registration process is lengthy. This reduced the students' interest and prompted them to choose to install the software in their devices. Having to install the software was considered a disadvantage (**D**_**7**_) for 4.4% of the students. Both the use of the free online version and the downloading of the software on devices was considered a threat that could constitute a reason for exclusion (**A**_**1**_) by 12.8% of the students. It means they need to have the appropriate devices as well as good internet connection, as pointed out by different groups: “*The online version posed problems, we had to install the programme to be able to work ... unwanted features were installed”* (Gr21) or: *“I couldn't use SketchUp from my tablet… not all of us have a good internet connection or computers*” (Gr7).

We analyse the results obtained below to interpret how the students perceived the use of the SketchUp software. The aim is to define the added value the use of the software has provided the workshop with for the understanding of the SGD according to the students, as well as to identify the obstacles and difficulties that arose from its use.

The strengths and opportunities the students pointed out in their responses related to the use of the software, teaching management and mathematical knowledge will help us interpret the reasons why the students believe the use of SketchUp is an added value for the implementation of the workshop and for the understanding of the SGD.

Although it is not clearly reflected in their answers, according to the data shown in Table 2, the students assess the teaching management of the workshop as positive, and a degree of satisfaction of 61.3% is observed.

The strengths and opportunities are grouped together in the table below. They include the elements of the software that facilitated its use, such as the potential uses of the software with regard to learning the SGD throughout the workshop (Table 4), as well as the frequency of mention (n_i_), and the percentage distribution.

**Table 4:** Categories and indicators associated with the high satisfaction of the use of the software.

Strengths, associated with:	n_i_	%
**C1**. S**oftware management**	77	-
**F**_**1**_. Intuitive, easy	77	37.9
**C2. Student motivation and involvement**	130	-
**F**_**2**_. Build the school in 3D in a realistic manner	41	20.2
**F**_**3**_. Contemplate the school built in 3D	12	5.9
**F**_**4**_. Become aware of the educational impact on the space	8	3.9
**F**_**5**_. Enhance the design by adding details	15	7.4
**F**_**6**_. Facilitate error identification	19	9.4
**F**_**7**_. Allow free experimentation	14	6.9
**F**_**8**_. Encourage creativity	21	10.3
**C3**. **Spatial understanding**	33	-
**F**_**9**_. The 3D representation stimulates structuring the space constructed	14	6.9
**F**_**10**_. Generates the need to use referential systems	19	9.4
**C4**. **Geometric understanding**	105	-
**F**_**11**_. Enables looking at the design from multiple angles	25	12.3
**F**_**12**_. Enables visualising how a geometric figure is formed	8	3.9
**F**_**13**_. Allows coordinating 2D and 3D representation	31	15.3
**F**_**14**_. Modifying the dimensions of a geometric figure helps identify its characteristics	17	8.4
**F**_**15**_. Helps building examples related to what has been covered in theoretical classes	24	11.8
**C5**. **Mathematical communication**	34	-
**F**_**16**_. Promotes dialogue, debate and negotiation that involve the use of topological, projective and Euclidian relations	19	9.4
**F**_**17**_. Promotes dialogue, debate and negotiation that involve the use of geometry	15	7.4
**Opportunities, associated with:**	**n**_**i**_	**%**
**C7. Innovations in the area of mathematics**	75	-
**O**_**1**_. Use the software to promote identifying and recognising plane figures and their properties	21	10.3
**O**_**2**_. Use the software to promote spatial orientation	17	8.4
**O**_**3**_. Use the software to coordinate 2D and 3D representation	37	18.2
**C8. Professional development**	64	-
**O**_**4**_. Design original didactic resources	41	20.2
**O**_**5**_. Promote the development of digital competence	23	11.3

It is observed that the most mentioned strengths correspond to *student motivation and involvement* (C2), to *geometric understanding* (C4) and to *software management* (C1), while the most mentioned opportunity corresponds to *innovations in the field of mathematics* (C7).

It can be seen that the most mentioned strengths correspond to the *intuitive and easy* indicators (**F**_**1**_), referring to the category of *software management* (**C1**), and *build the school designed in 3D in a realistic manner* (**F**_**2**_), which belongs to the *student motivation and involvement category* (**C2**). The most mentioned opportunities, on the other hand, correspond to the indicators *Design original didactic resources* (**O**_**4**_), referring to the *professional development* category (**C8**), and *use the software to coordinate 2D and 3D representation* (**O**_**3**_), which belongs to the category *Innovations in the area of mathematics* (**C7**).

With reference to the software management (**C1**), 37.9% comments that the use of SketchUp is easy and intuitive (**F**_**1**_), as shown in the statement of one of the groups: “*We did not need to see any tutorial to use SketchUp, it is easy to use*” (Gr15). This fact contrasts with the perception of 41.4% of the students that considers its use is complicated and difficult (**D**_**1**_).

Regarding student motivation and involvement (**C2**), the experiment of completing the workshop with the construction of a tangible product, their ideal school realistically represented in 3D (**F**_**2**_) is a reason for great satisfaction for 20.2% of the students, as evidenced in the statements of some of the groups: “*Capturing what would be our ideal school in a sketch has been an interesting challenge, bringing our school to life and contemplating it in 3D has been a very rewarding experience*” (Gr26) or “*We never imagined that we would be such good architects and that it would be fun to do*” (Gr17).

The experience of the 3D modelling process using the school software for shaping, organising spaces, decorating and adding significant details (**F**_**5**_) (furniture, areas for free play, exteriors, etc.) as well as the final experience of contemplating the built school, moving around in it, observing its form, organisation, decoration, etc., (**F**_**3**_) was pointed out by 7.4% and 5.9% of the students as a very satisfactory experience. One of the groups said the following: “*We spent a lot of time adding details to our school… looking at the equipped and decorated classrooms provides the design with a lot of meaning and makes the effort worthwhile*” (Gr31).

**Figure 5:**
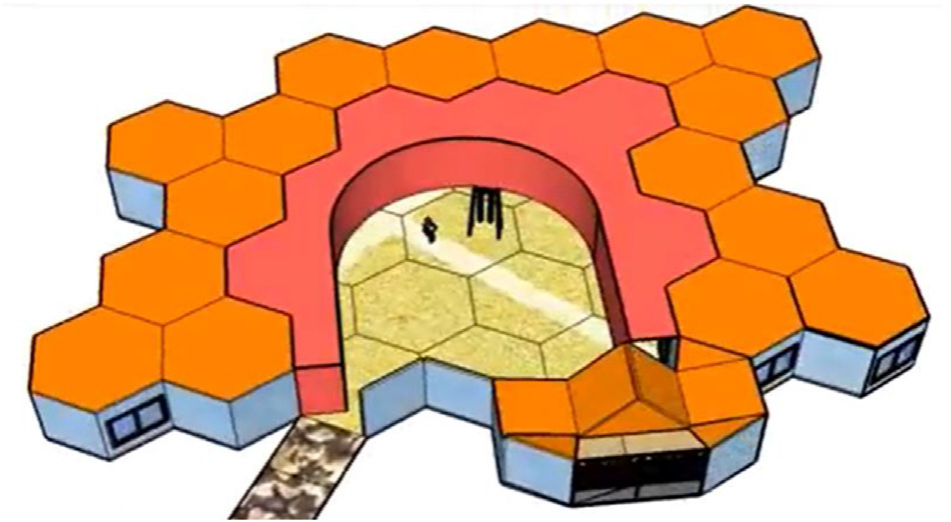
Design of a school inspired by a beehive (Gr5).

It should be noted that for 6.9% of the students the use of the software led to free experimentation (**F**_**7**_) and 10.3% considered it a stimulating experience to encourage creativity (**F**_**8**_) (Figure 5). No less striking is that, for 3.9% of the students, the importance of the workshop, and specifically the possibility of making decisions and executing them through software, made them become aware of the educational impact on the space (**F**_**4**_). The following statement by one of the groups reflects the previous comments: “*Between the first sketch we made to the final school, there is a world of distance… we were highly motivated imagining what the school we would like to work at would look like… A lot of decisions need to be made and many details need to be thought of ... The school also educates!*” (Gr5).

As far as the understanding of space and geometry (**C3** and **C4**) is concerned, 15.3% of the students comments the software facilitates the knowledge and development of the transition from 2D to 3D (**F**_**13**_), and, as indicated by 3.9%, it enables visualising how a geometric figure is formed (**F**_**12**_). Together with the possibility of modifying the dimensions of geometric figures, this was pointed out by 8.4% as an aspect that helps identifying the characteristics of both plane figures and geometric shapes (**F**_**14**_), as one of the groups says: “*It helped us elevate the plans we were designing, and change what seemed inappropriate*” (Gr28).

6.9% of the students consider as a strength of the software its potential to work on different concepts such as spatial structuring (**F**_**9**_), and the establishment of projective relationships (**F**_**10**_). As is observed, 12.3% of the students stated that it enables visualising the designed elements (figures or shapes) from numerous angles (side, top, front, etc.). One of the groups commented: “*From the first try to the last, we managed to improve the views, from different places of the parts to be built, and to better adjust the designs*” (Gr29).

**Figure 6:**
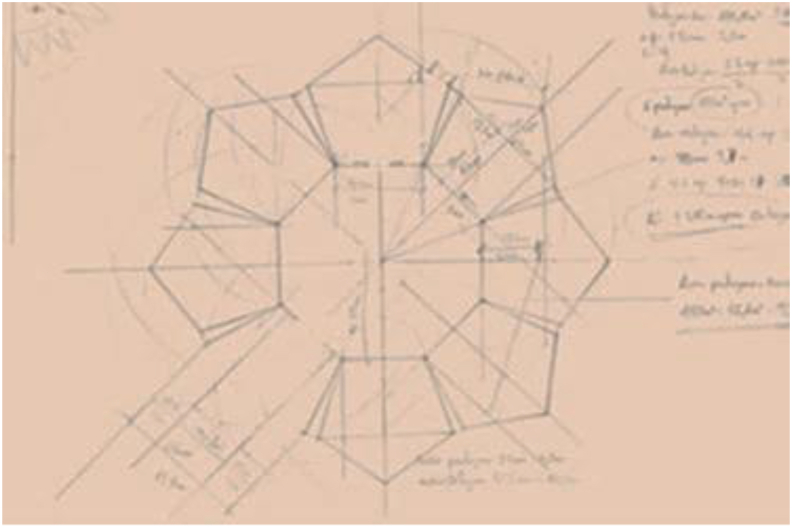
Sketch of the design of a school by a group (Gr11).

Another strength they consider this software has is the possibility of measuring lengths or angles to check their design and the measurements they thought were appropriate, as is the case shown in Figure 6. Starting from a sketch in which there is no connection or an overlap between different rooms in some parts, the software itself served as a corrective element (**F**_**6**_) to discern whether to use regular or irregular pentagons or not (**F**_**17**_). This was pointed out by one of the groups: “*When modelling the floor plan of the school with SketchUp, we realised that, in order not to generate gaps between the rooms, we had to use irregular pentagons*” (Gr11). 9.4% of the students regards the software as an important element that facilitates identifying errors (**F**_**6**_) related to the measurements.

Finally, with regard to how the use of the software generates new opportunities to build or strengthen knowledge of space and geometry, it is worth mentioning the use 11.8% of the students made of the software to define new examples to illustrate the contents worked on previously (**F**_**15**_). An example is visualising the different types of borders (open, closed, continuous, discontinuous, adjacent, etc.) linked to the different topological relationships established between the spaces and elements of the school. One of the groups indicated: “*To connect the parts of the school, we created mobile parts to connect or separate rooms*” (Gr24).

The dialogues, debates and negotiations that inevitably took place in the small working groups around the mathematical problems of form and position in the design and 3D modelling of the school involved the use of topological, projective and Euclidean (**F**_**16**_) relationships. This is stated by 9.4% of the students, and mentioned by one of the groups: “*In our proposal, we observe that distance and proportion are very important*” (Gr8).

The opportunities perceived by the students are in line with the strengths encountered. As to the possibility of using the software for didactic innovations in the area of mathematics (**C7**), the possible didactic use most observed by the students (18.2%) concerns facilitating the coordination between 2D and 3D representations. 10.3% of the students recognises the potential educational use of the software to promote identifying and recognising plane figures and their properties (**O**_**1**_), while 8.4% does so with an eye toward enhancing spatial orientation (**O**_**2**_). One of the groups said: “*It helped us use the sides of the figures and their appearances the way we wanted to orientate them*” (Gr25).

Lastly, it should be noted that the final open question of the Likert questionnaire, the purpose of which was to enable students to express other opinions of interest, revealed that 14% of the students considered the correct completion of the workshop required knowing much more geometry than the basic knowledge they gained from previous educational experiences. This reflects an obvious weakness in mastering the knowledge of reference, the space and geometry domain.

## 6 Discussion

The most significant results will be discussed below. First of all, the potential the students perceive in the software to facilitate the understanding of the SGD will be stressed. The main reasons that conditioned the degree of satisfaction of the students with regard to the use of the software will then be discussed. Finally, the main limitations of the study will be presented.

### 6.1 Student perceptions of the potential of SketchUp to facilitate the understanding of the SGD

The results obtained show that the use of the SketchUp software has facilitated the students' understanding of the SGD in a manner similar to what other authors who have used the same software have pointed out (Dolenc and Aberšek, 2012; Panorkou and Pratt, 2016; Uygan and Kurtuluş, 2016; Nurwijayanti et al., 2019). Amongst the most significant results is the fact that the students state the software has facilitated their understanding of the transition from 2D to 3D, allowing them to identify the characteristics of both plane figures and geometric shapes.

The software does not enable generating 3D shapes using a button. To create a 3D shape, there are two possibilities: starting from a 2D shape or modifying a previously constructed 3D shape. This situation, which in other disciplines could be interpreted as a disadvantage or a lack of software functionality, generates an extremely important transition that facilitates the understanding of the change from 2D to 3D.

Another noteworthy aspect of the results regarding the understanding of the SGD is that the features of the software to measure lengths and angles helped the students correct previously elaborated 2D sketches.

Finally, it should be noted that the students perceived the use of the software allowed them to consolidate content previously worked on during the course, such as the topological, projective and Euclidean relationships shown in the different types of borders, different perspectives, and the lines and angles that define the limits of the rooms.

What the students perceived coincides with what Soto Varela (2014) pointed out about the software. According to him, it facilitates drawing in two dimensions on a plane and then enables giving it volume using the third dimension. It immediately allows having 2D views (elevation, plan, profile, etc.) of the 3D figure that is being modelled. The software also allows measuring dimensions and angles, using and changing scales, and even adding annotations on the figure in both 2D and 3D. The free library of objects, pictures and textures can be used.

Regarding the ability of the software to help improve visual and spatial skills (Kurtulus and Uygan, 2010; Toptaş et al., 2012; Dolenc and Aberšek, 2012; Turgut and Uygan, 2015; Chou et al., 2017; Wahab et al., 2018; Williams and Capraro, 2020; Šafhalter et al., 2020; Jaelani, 2021), the students perceived the possibility of building 3D shapes, modifying their dimensions and visualising them from a multitude of perspectives as a strength that allowed improving skills related to spatial orientation and visualisation.

### 6.2 Degree of student satisfaction regarding the use of the software

As far as the low satisfaction of the students regarding the use of the software is concerned, one of the most significant reasons is related to its manageability. Although most research studies point out that its use can be learnt quickly and easily (Erkoç et al., 2013; Chou et al., 2017), the results obtained in the present study reveal that only 37.9% of the students had this perception. In contrast, 41.4% considered that its use is complicated and expensive, since it requires time to reflect and practise, a fact that coincides with the results provided by Uygan and Kurtuluş (2016).

Studies carried out by Dolenc and Aberšek (2012) and Lee and Kim (2013) show that work environments in the classroom are one of the advantages generated by the use of the software. Non-face-to-face classes and the alternation of synchronous and asynchronous classes constitute a unique educational context that has had an impact on the work environment. The results of our study reveal that both the lack of immediate feedback from the teacher with regard to meeting the needs related to the management of the software (18.2%), and the difficulty of working from home without direct contact with their peers (11.8%) negatively affected the students' involvement and motivation as far as the use of the software was concerned.

The impossibility of using all the software features with the free version (12.8%), together with the students' perception that there are other programmes (Second life, The Sims, SimCity) that are easier to use and provide more possibilities to the designs (27.1%) is another important reason linked to the low student satisfaction with respect to the use of the software.

The results obtained reveal that the high student satisfaction regarding the use of the software is basically because the software enables creating realistic designs (Ramadhanty and Handayani, 2020). The possibility of providing their ideal school with significant details was perceived as a stimulus during the 3D modelling process (7.4%), and contributed to encouraging the students' creativity (10.3%).

### 6.3 Study limitations

During the implementation of the workshop, the work of the groups was monitored through virtual meetings. In these meetings, the students shared their progress regarding the design of their ideal school, as well as their difficulties related to managing the software. In order to make sure that all the students used the software, questions were asked to the different members of the groups regarding its use. On the whole, the information provided by the students in these meetings was consistent with the statements subsequently collected in the two questionnaires. We believe this proves the veracity of the students' statements. The main limitation of this study was not to be able to carry out an individual follow-up of each of the 203 study participants with regard to the use of the software. As the responses to the two questionnaires were collected from the different groups, we cannot rule out the possibility that one or more students did not use the software or hardly invested any time in its use.

## 7 Conclusions

The pandemic that affects us globally has turned the use of new technologies for teaching into an essential tool to maintain contact between students and teachers. Taking advantage of the opportunities offered by new technologies, González-González et al. (2019) point out they can be used to fulfil the teaching objectives in the teaching and learning process, despite the students' doubts or reluctance. The use of virtual platforms such as Google Meet, as in this case, or Skype, Teams or Zoom help creating a feeling of closeness between teaching participants, students and teachers. They allow debating on the concepts or notions worked on in the training workshop, thus enriching the final result of both the subject and the design and construction of the students' ideal school.

The final result of the workshop, the students' design of their ideal school, including principles of teaching and learning, allowed them to work on the interior and exterior of the different buildings or rooms of their ideal school within the space and geometry domain. Using the SketchUp software, they started from geometric figures in 2D, and converted them into 3D models, thus working on the two perspectives. Likewise, the different spatial relationships in each of the rooms were worked on in a more practical and clarifying way than in the theory classes, as the students pointed out in their comments.

To conclude, it may be said that the students had a positive perception both of the workshop and of the use of the software. They were able to strengthen their own knowledge of the SGD through the development of digital competence (Casillas Martín et al., 2019) using SketchUp, a 3D design software. The effectiveness of the use of technology in education should always be considered contingent and depends on how and in which context it is used (Mahmud, 2018).

The results referring to both the perception of the potential of SketchUp to facilitate the understanding of the SGD, and the degree of student satisfaction regarding its use provide us with clues on how to create a new design that considers strengths and opportunities, and reverts the weaknesses and threats observed.

## Declarations

### Author contribution statement

Enrique Carmona Medeiro: Conceived and designed the experiments; Performed the experiments; Analyzed and interpreted the data; Contributed reagents, materials, analysis tools or data; Wrote the paper.

Juan Antonio Antequera Barroso and José María Cardeñoso Domingo: Performed the experiments; Analyzed and interpreted the data; Contributed reagents, materials, analysis tools or data; Wrote the paper.

### Funding statement

This research did not receive any specific grant from funding agencies in the public, commercial, or not-for-profit sectors.

### Data availability statement

Data will be made available on request.

### Declaration of interests statement

The authors declare no conflict of interest.

### Additional information

No additional information is available for this paper.
